# Rational Structural Design of Polymer Pens for Energy-Efficient Photoactuation

**DOI:** 10.3390/polym15173595

**Published:** 2023-08-29

**Authors:** Zhongjie Huang, Le Li, Taishan Yin, Keith A. Brown, YuHuang Wang

**Affiliations:** 1 State Key Laboratory for Modification of Chemical Fibers and Polymer Materials, College of Materials Science and Engineering, Donghua University, Shanghai 201620, China; 2 Department of Mechanical Engineering, Boston University, Boston, MA 02215, USA; 3 Department of Chemistry and Biochemistry, University of Maryland, College Park, MD 20742, USA; 4 Maryland NanoCenter, University of Maryland, College Park, MD 20742, USA

**Keywords:** photoactuation, PDMS, elastomeric microstructure array, crosstalk, actuation efficiency

## Abstract

Photoactuated pens have emerged as promising tools for expedient, mask-free, and versatile nanomanufacturing. However, the challenge of effectively controlling individual pens in large arrays for high-throughput patterning has been a significant hurdle. In this study, we introduce novel generations of photoactuated pens and explore the impact of pen architecture on photoactuation efficiency and crosstalk through simulations and experiments. By introducing a thermal insulating layer and incorporating an air ap in the architecture design, we have achieved the separation of pens into independent units. This new design allowed for improved control over the actuation behavior of individual pens, markedly reducing the influence of neighboring pens. The results of our research suggest novel applications of photoactive composite films as advanced actuators across diverse fields, including lithography, adaptive optics, and soft robotics.

## 1. Introduction

**Figure 1:**
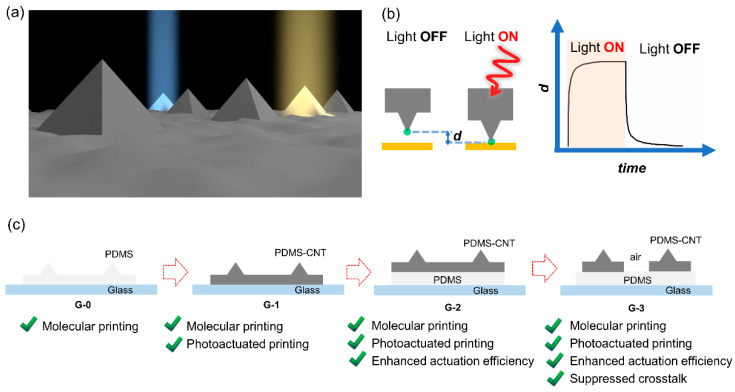
Novel structural designs of photoactuated polymer pen arrays. (**a**) Scheme illustrating the concept of addressing individual units in a massive elastomeric microstructure array for site-selective actuation and multiplexed sensing for various applications. (**b**) Scheme showing the operation mechanism of pPPL in which the pen’s vertical movement is controlled by illumination. The photoactuation magnitude is characterized by the vertical extension *d* of the photoresponsive film in the *z* direction. (**c**) Rational design of new generations (G1–G3) of pen array structures built off the original polydimethylsiloxane (PDMS) PPL pens (G-0). The red arrows show the evolution process. The materials shown for the films on the glass substrate (blue) are either pure PDMS (light grey) or light-absorbing PDMS-carbon nanotube (PDMS-CNT) composites (dark grey). The added function in each design is highlighted.

Elastomeric microstructure arrays, consisting of matrices of regularly arranged microstructures made from elastomeric materials, have garnered increasing interest in various domains, including flexible electronics [1,2], sensing [3,4,5,6], soft lithography [7,8], biomimetic surfaces [9,10,11,12,13], soft actuators, and robotics [14,15,16]. Their unique characteristics, such as reversible deformation under force, a high specific-area contact surface with adjustable physical and chemical properties, potentially parallel addressability with high resolution, and active structural responses to electromagnetic waves, make these systems a diverse platform for functions including patterning materials [17,18], directing chemical reactions, transferring energy [19,20,21], and collecting signals [22]. The simultaneous use of stimuli-responsive fillers or coatings further enhances the versatility of such a platform [3,9,23,24,25,26], enabling multiple actuating and sensing capabilities (refer to Figure 1a). The standout feature of these arrays, however, is the ability to direct the activity of individual elements within the arrays [25]. This capability opens doors to site-specific administration, high-throughput actuation and sensing, and material manipulation. Notably, this capability has been demonstrated in cantilever-free scanning probe lithography [17,19], wherein arrays of pyramidal polymer pens are used to achieve multiplexed molecular printing [20,21,27,28]. Thus, it is possible to transform elastic microstructure arrays into a general micro/nanoscale platform with high throughput and low cost.

Despite remarkable progress in various aspects of microstructure functionality, the pursuit of local actuation with high spatiotemporal accuracy, precision, and energy efficiency remains a captivating challenge in the realm of micro/nanotechnology. This challenge stems from fundamental inquiries and the vast potential application possibilities in fields such as programmable materials, micro/nano robotics, haptic rendering, and precision medicine. While considerable efforts have been devoted to understanding the effect of microstructure on surface hydrophobicity/hydrophilicity [29], adhesiveness [30,31,32,33], anti-icing capability [34], optical properties [35], and the mechanical sensitivity of devices [3,4], limited attention has been paid to its influence on the local actuation behavior and energy regulation, i.e., the fraction of energy utilized for actuation relative to the energy dissipated into the environment. To achieve precise and efficient local actuation within a micro-system, it is essential to understand the conversion and dissipation pathways of heat and energy across different microstructures.

Photoactuated polymer pen lithography (pPPL) allows remote and dynamic control of select pens in a massive array for ink writing through material transport [28,36]. This capability is significant for arbitrary nanomanufacturing and nanopatterning with high throughput and low cost. In this system (as shown in Figure 1b), the vertical motion of selected pens is controlled by illumination. When illuminated, the light-absorbing film expands along the *z*-axis, moving the pen towards the substrate and enabling ink transfer. Conversely, deactivating the illumination causes the pen to contract, effectively halting the writing process. Previous light-coupled atomic force microscope (AFM) and printing experiments have consistently demonstrated the fast (with time constant at sub-1 s timescale) and reversible nature of the photoactuation [28,37], enabling stable and high-quality patterning. In this system, the actuation efficiency (AE) is defined as AE = *d*/*P*, where *d* is the out-of-plane deformation of the film in the *z* direction (Figure 1b), and *P* refers to the total input energy delivered to the region corresponding to a single pen [28]. Despite significant progress [38], achieving actuation at single pen resolution remains a significant challenge. Commensurate efforts are required from both materials science and device engineering to tackle this problem. In this study, we introduce novel generations of photoactuated polymer pens with rationally designed structures (Figure 1c) and examine their photoactuation capabilities for multiplexed nanolithography through both simulations and experiments. We show that incorporating a thermal insulating layer and airgaps has doubled the photoactuation efficiency and significantly suppressed thermal crosstalk. By diminishing the influence of neighboring counterparts, pens within a massive array can be separated into isolated units for efficient energy utilization and mechanical actuation.

## 2. Materials and Methods

**Figure 2:**
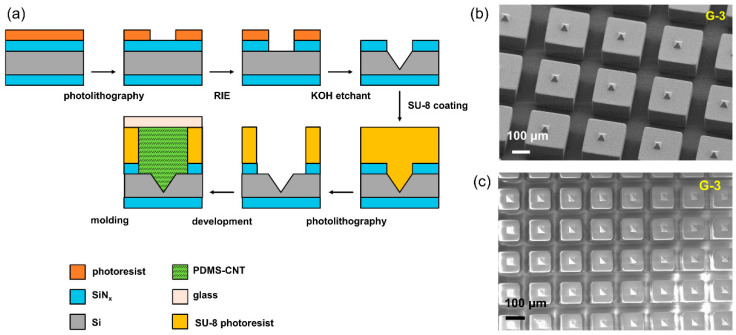
Airgap-isolated polymer pens. (**a**) Scheme showing the fabrication process used to realize the airgap-isolated polymer pens using a two-step lithographic approach to fabricate the master. The scanning electron microscopy (SEM) images of the pens with defined pitch distance: (**b**) 250 µm; (**c**) 150 µm.

Pen array fabrication: The preparation of PDMS-CNT paste can be found in our previous studies [28,38]. The pen arrays with G1-3 structures were produced using corresponding master molds. Multiwalled CNTs (SWeNT^®^ SMW 100) were covalently grafted with long alkyl chains –(CH_2_)_5_CH_3_ [38] and used as light absorbers in the PDMS composite film. For the G-2 pens, a PDMS film was layered between the glass substrate and the PDMS-CNT light-absorbing layer, with the thickness being determined by a profilometer (Tencor Alpha Step 200). Following this, the uncured PDMS-CNT composite paste was poured onto the conventional Si master with pyramidal cavities. The glass substrate containing the PDMS insulation layer was positioned on top, and the system was cured at 80 °C for 12 h. For the G-3 pens, the Si-SU-8 master, as shown in Figure 2a, was fabricated using a two-step lithography approach. In the initial lithography step, pyramidal holes were created through the conventional anisotropic etching of silicon using a KOH solution. The subsequent lithography step added the SU-8 walls onto the Si wafer, completing the desired template. A coating of fluorinated silane (heptadecafluoro-1,1,2,2-tetra(hydrodecyl)trichlorosilane) was applied to the master for easy release. Films and pens were fabricated using parameters congruent with those used in simulations. Specifically, for the G-3 design, a pen array consisting of an 80 µm thick backing layer, with a 150 µm pitch, 120 µm pillar edge, and 200 µm pillar height, was used for investigation.

Simulation: Finite element analysis (FEA) simulations were conducted using commercially available software (COMSOL 5.3a, COMSOL Inc., Stockholm, Sweden). Building upon prior discussions [28,37], the PDMS-CNT film’s out-of-plane deformation under illumination was effectively explained by the composite’s thermomechanical properties. The analysis modeled the thermomechanics of a PDMS film on a glass side with a central heating region. To replicate the heating from absorbed light, an exponentially decaying heating intensity derived from Beer–Lambert law, which describes light attenuation through partially absorbing materials, was employed along the vertical direction of the film. Specifically, the incident intensity *I*(z) = *I*_0_ exp(−z/z_0_) diminishes exponentially with a characteristic length z_0_, which depended on the concentration of CNT in the PDMS film. To ascertain z_0_, we found that absorbance was 95% across the film thickness of 200 µm. This information allowed the calculation of the locally absorbed power density as *I*_0_ exp(−z/z_0_) by taking the z-derivative of the light intensity.

To account for heat dissipation into the surrounding environment, free convective heat transfer boundary conditions were applied to the interfaces exposed to air. Additionally, a fixed mechanical boundary condition was applied on the bottom of the supporting glass slide. The PDMS-CNT composite used in the analysis had a significantly low CNT concentration of only 0.25%. Consequently, the physical properties of the composite, including thermal conductivity, heat transfer coefficient, and Poisson’s ratio, were predominantly governed by PDMS, as illustrated in the previous study [28,37]. Hence, the material properties of PDMS were directly assigned to the composite in FEA simulations (see Table 1). To capture the deformation dynamics of the PDMS-CNT film in response to incident light, all simulations were conducted using a time-dependent approach. Given that the dwell time of the polymer pen during patterning typically operates on a second-scale timeframe, the simulation investigations were strategically confined to the initial five-second deformation trajectory of the film.

AFM characterization: The photoactuation of the pens was investigated using AFM (MFP-3D Infinity, Asylum Research). To locally illuminate the sample from beneath, a broad-band LED (MCWHF2, Thorlabs, Newton, NJ, USA) was integrated into the base of the AFM system. The AFM probe was positioned on the tip of the pen, and the illumination area around the probe was confined to a radius of *R* by means of a pinhole in a 100 nm thick aluminum film on a glass slide. The pinhole was fabricated by directly depositing aluminum onto a pre-cleaned glass using an electron beam evaporation system, and *R* was chosen to be 125 µm.

During the experiment, the LED was periodically activated using a function generator, while the AFM was scanned in tapping mode with an extremely small imaging area (10 × 10 nm^2^) at the slowest scanning rate possible (0.1 Hz). This setup ensured that the AFM tip remained nearly static in the *x* and *y* directions, while the feedback retained contact with the fluctuating pen. By alternating the illumination on and off, the AFM topographical images could be translated into surface trajectories, enabling a detailed study of the pen’s photoactuation behavior.

## 3. Results and Discussion

As shown in Figure 1c, a series of distinct pen arrays were designed and fabricated. The rationale behind the design is based on enhancing the complexity of the pen structure by introducing specific added functions. Generation 0 (G-0) shows the conventional elastomer pen array used in soft lithography, which consists of an array of polymer microstructures (e.g., pyramids) that rest on an elastomeric film on a rigid backing substrate (e.g., glass). The most used polymer for PPL is Sylgard 184 PDMS, which is employed in this work. To enable photoactuation, a photoresponsive composite (PDMS-light absorber nanocomposite) is adopted to transform the pen array into a photoactuable system. This transformation is represented by G-1. In our previous studies, we have shown that short CNTs are promising candidates for light absorbers in photoactuated PDMS. This is due to the following reasons: (i) CNTs, unlike most organic chromophores, are inert to the Si-H moiety of the PDMS crosslinker, which negates the inhibition of PDMS crosslinking; and (ii) chemical reactions for surface grafting significantly enhance the dispersion of CNTs within the PDMS matrix. This results in the creation of a uniform and transparent film that also exhibits effective light-absorbing properties. This renders the film suitable for applications involving photoactuation and lithography [38]. Consequently, CNTs were selected as the light absorber in this study.

Given the significantly higher thermal conductivity of glass (1.0–1.4 W·m^−1^·K^−1^) compared to PDMS (0.15–0.25 W·m^−1^·K^−1^), we designed a novel pen architecture (G-2), where a PDMS backing layer was inserted between the light-absorbing layer and the glass substrate. This PDMS layer serves as a thermal insulator that effectively minimizes the energy loss through the substrate. Contemplating the thermal conductivity of air (0.024 W·m^−1^·K^−1^)—an order of magnitude lower than PDMS—we innovated a further iteration of the pen architecture (G-3), in which each pen on the light-absorbing layer was separated by an airgap. This approach holds great potential in mitigating crosstalk while retaining all the advantages presented by the previous designs.

Fabricating G-2 proved straightforward—by depositing a PDMS layer onto the glass substrate, followed by the fabrication of a pyramidal pen array using a pre-machined silicon master. However, the realization of G-3 required careful design, utilizing a two-step lithography approach delineated in Figure 2a. In this protocol, the first lithography step resulted in creating pyramidal holes via the standard anisotropic etching of silicon using a KOH solution. Subsequently, the second lithography phase comprised the addition of SU-8 walls onto the silicon wafer, thereby resulting in the desired template. This template was utilized in the fabrication of G-3 pens. As evidenced in the scanning electron microscope (SEM) images shown in Figure 2b,c, we successfully prepared G-3 pens with pitch distances of 250 µm and 150 µm, respectively, demonstrating clear airgaps between the pillars, hence rendering each pen as an independent unit.

**Figure 3:**
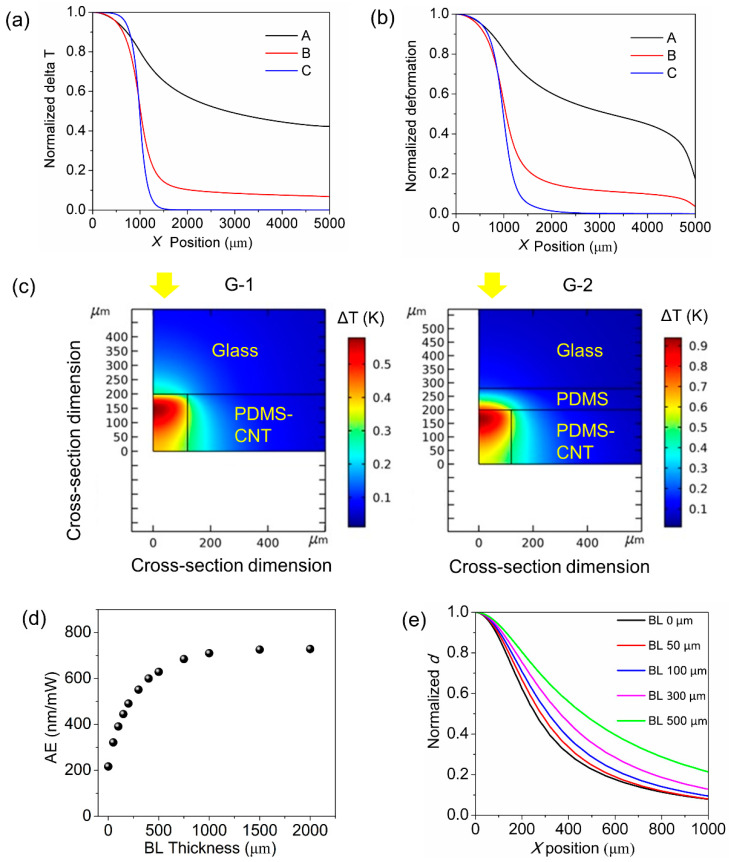
Photoactuation simulations of G-1 and G-2 pens. (**a**,**b**) Spatial distribution of normalized parameters for G-1 pens (200 µm thick light-absorbing layer with an illumination radius of 1000 µm) with variable elastomer thermal conductivity (sample A: 0.25 W·m^−1^·K^−1^; B: 2.5 × 10^−2^ W·m^−1^·K^−1^; C: 2.5 × 10^−4^ W·m^−1^·K^−1^): (**a**) top surface temperature change; (**b**) deformation. (**c**–**e**) Photoactuation simulation of G-2 pens (200 µm thick light-absorbing layer, 80 µm thick PDMS backing layer, illumination intensity of 450 mW cm^−2^ with an illumination radius of 125 µm). (**c**) Surface plot showing the temperature distribution for G-1 and G-2 pen architectures. The yellow arrows indicate the location of the illumination beam on the back of the glass, mimicking the pPPL operation (pens are facing down). The dependence of photoactuation efficiency on the backing layer (BL) thickness at an illumination time of 5 s: (**d**) at the illumination center, *x* = 0; (**e**) normalized deformation along the film.

A thorough evaluation of the photoactuation capabilities possessed by pens with varied architectures (G1-3) was undertaken through the combined use of simulations and experiments. As depicted in Figure 3, a series of finite element analysis (FEA) simulations was performed to understand the origin of crosstalk and the impact of the PDMS backing layer on photoactuation efficiency. In a previous study, we showed that the photoactuation behavior of the polymer pens can be explained by the local photothermal effect [28]. However, the underlying mechanism of the crosstalk effect remains unverified. Therefore, the spatial distribution of the deformation of PDMS materials was simulated to examine the role of the material’s thermal conductivity in contributing to crosstalk. The fundamental notion is that hypothetically altering the thermal conductivity (Sample A: 0.25 W·m^−1^·K^−1^; B: 2.5 × 10^−2^ W·m^−1^·K^−1^; C: 2.5 × 10^−4^ W·m^−1^·K^−1^) of the elastomer will influence the dispersal of heat (from light illumination with a radius of 1000 µm) across the film. Specifically, if the thermal conductivity is decreased, heat will be increasingly localized in the central area, resulting in a heat profile that abruptly terminates at the heating source’s boundary. If the deformation pattern remains unvaried with different thermal conductivities, then other factors, such as mechanical stress, must have instigated the crosstalk. Conversely, if the deformation pattern agrees with the thermal energy distribution pattern, the cause of the crosstalk should be mainly thermal. Our results, shown in Figure 3a (presenting the normalized temperature change vs. position) and 3b (presenting normalized deformation vs. position), reveal a substantial positive correlation, indicating that crosstalk is predominantly induced by the diffusion of heat. Notably, when thermal conductivity is set as 2.5 × 10^−4^ W·m^−1^·K^−1^ (sample C) and the delta temperature is zero at *x* = 2000 µm, deformation is still observable, suggesting that long-range mechanical stress exerts a minor influence over the system.

Having shown the importance of thermal diffusion, we explored architectures designed to mitigate this effect. To do this, the thermal conductivity of PDMS was maintained at 0.25 W·m^−1^·K^−1^ for the following simulations. The heat distribution of the photoactuated pens, as shown in the surface plots (Figure 3c), suggests that the inclusion of a PDMS backing layer (80 µm thick) between the PDMS-CNT light-absorbing film (200 µm thick with 0.25 wt.% of CNT) results in a nearly twofold increase in temperature at the hottest regions. Additionally, there is a reduction in the dissipated of thermal energy through the glass substrate. These findings confirm that the glass substrate constitutes a primary path for heat loss during the photoactuation process, and the PDMS thermal insulating layer significantly reduces heat dissipation through the glass. Moreover, the heat diffusing through the PDMS backing layer also adds to the overall photoactuated deformation.

While the incorporation of the thermal insulating layer has been proven effective, its thickness can potentially influence the photoactuation efficiency and crosstalk between neighboring pens. A series of simulations were conducted in which the thickness of the backing layer was varied while the illumination radius was maintained at 125 µm and the light-absorbing film thickness was fixed at 200 µm. The simulations show that the photoactuation efficiency of the pens increases with the thickness of the backing layer, suggesting that a thicker thermal insulating layer allows the energy to be transduced to actuation more efficiently. However, upon reaching a thickness of 1 mm, the actuation magnitude plateaus (refer to Figure 3d), indicating saturation in the impact of thermal insulation. Consequently, crosstalk exhibits a gradual rise with increasing backing layer thickness (refer to Figure 3e). Thus, an 80 µm thick backing layer was selected as the optimal configuration for the G-2 architecture.

**Figure 4:**
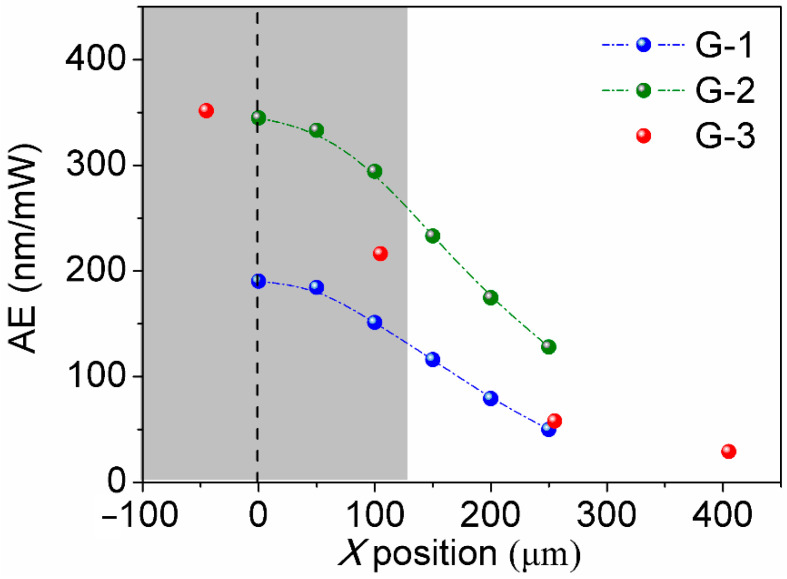
Photoactuation efficiency of G-1, G-2, and G-3 pen architectures (blue, green, and red dots, respectively) examined by light-coupled AFM characterization. The grey block shows the illumination area (with a radius of 125 µm). The blue rectangles show the positions of the pillar unites separated by the airgaps in the G-3 structure.

The actuation capability of pens with G-1, G-2, and G-3 structures was studied using light-coupled atomic force microscope (AFM) experiments [28]. For the G-3 design, a pen with an 80 µm thick backing layer, 150 µm-pitch, 120 µm pillar edge, and 200 µm pillar height was used. As presented in Figure 4, the incorporation of an 80 µm thick PDMS backing layer within the structure (G-2) nearly doubles the photoactuation efficiency of the pens (as shown by comparing the green and blue dots), which agrees well with the simulation results. To evaluate the crosstalk effect, comparisons were drawn between actuation magnitudes at a distance *x* away from the illumination center and those at the illumination center (*x* = 0 µm). The illumination radius was 125 µm, realized using a pinhole mask. It is noted that precise alignment of the illumination center with the pillar’s center point in both *x* and *y* directions is significantly challenging in light-coupled AFM experiments; thus, a slight deviation from zero for the central illumination point is noticeable. Figure 4 elucidates that G-3 (red) suppresses crosstalk expansion (at *x* = 250 µm) to a value below 15% of the central expansion, while G-2 without an airgap exhibits 37% crosstalk. In addition, G-3 effectively suppresses crosstalk expansion at *x* = 400 µm and limits it to under 8% of the central expansion. Simulated G-1 photoactuation, on the other hand, shows more pronounced crosstalk that reaches 30%. This demonstration unambiguously shows that the innovative pen array architecture of G-3 is capable of concurrently doubling the actuation energy efficiency while reducing thermal crosstalk.

## 4. Conclusions

In summary, we observed a significant impact of pen architecture on the photoactuation efficiency and crosstalk. Through modifications to the pen array structure, we achieved enhanced local photoactuation with remarkable spatial resolution, while effectively mitigating the crosstalk between neighboring pens. This advance was made possible by isolating the pens into discrete units, accomplished by integrating an airgap and embedding a thermal insulating layer. The implications of our work extend to diverse research areas, including polymer composites, smart materials, actuators, lithography, and soft robotics, making it highly relevant and promising for further advancements in these fields.

## Figures and Tables

**Table 1:** Properties of the PDMS-CNT films utilized for the FEA simulations.

Property	Value
Thermal expansion coefficient (α)	2.99 × 10^−4^ K^−1^
Thermal conductivity (*k*)	0.25 W·m^−1^·K^−1^
Young’s modulus (*E*)	750 kPa
Poisson’s ratio (*υ*)	0.49999

## Data Availability

The data are available in the manuscript.
